# Public health in Sweden during the COVID-19 pandemic: Anna Månsdotter

**DOI:** 10.1093/eurpub/ckac129.005

**Published:** 2022-10-25

**Authors:** A Månsdotter, G Fochsen

**Affiliations:** 1 Living Conditions and Lifestyles, Public Health Agency of Sweden, Solna, Sweden; 2 Department of Epidemiology and Global Health, Umeå University, Umeå, Sweden

## Abstract

**Background and objectives:**

The COVID 19 pandemic has highlighted how public health is dependent on many areas of society, and several aspects of public health can be affected. We have evaluated how the COVID-19 pandemic and the measures taken to reduce its spread have impacted public health in Sweden during 2020.

**Methods:**

We systematically compiled international research on the pandemic's impact on public health, we examined living conditions of groups at a particularly increased risk of ill health, and we collected and analysed Swedish data on lifestyles, health, injury, and illness during the pandemic compared to previous years.

**Results:**

Most people have, in one way or another, been affected by the pandemic and by societýs preventive measures. However, some groups have suffered more than others. Groups who were already at an increased risk of ill health before the pandemic have been most affected, e.g. in schools, on the labour market, and in society in general. There is a risk of increased health inequality, not only related to morbidity and mortality of COVID-19 during 2020, but also when it comes to the effects on living conditions.

**Conclusions:**

The consequences of the COVID-19 pandemic pose major challenges for public health, and the measures taken to limit its spread inter-relate with social and economic conditions. In Sweden, health inequalities have remained the same or increased over the years. Our study suggests that the consequences of the pandemic will reinforce health inequalities. It is too early to determine what the pandemic's full impact on public health will be. Nevertheless, health promotion and preventive measures need to be strengthened and prioritized in order to maintain good public health and reduce inequalities.

